# 

**Published:** 1903-09

**Authors:** 


					﻿t4tion $100.
jyMpjrSHEd MONTHLY
ATLANTA
JOURNAL-RECORD
OF MEDICINE.
Successor to Atlanta fledical and Surgical Journal, Established 1855,
and Southern fledical Record, Established 1870.
Vol. V.
Atlanta, Ga., September, 1903.
No. 6.
				

